# Electrochemical biosensors based on the 3D immobilization of capture probes for influenza virus detection

**DOI:** 10.1039/d5ra03744a

**Published:** 2025-08-12

**Authors:** Hyo Won Kim, Ae Sol Lee, Chang Sup Kim

**Affiliations:** a Graduate School of Chemical Engineering, Dongguk University Seoul 04620 Republic of Korea; b Department of Chemical and Biochemical Engineering, Dongguk University Seoul 04620 Republic of Korea biocskim@dgu.ac.kr

## Abstract

Influenza viruses pose a significant global health threat, particularly to vulnerable groups such as young children, the elderly, and individuals with underlying health conditions. Accurate and early detection is vital for effective disease management and the prevention of viral transmission. However, traditional diagnostic methods, including viral cultures, rapid antigen detection, and polymerase chain reaction, often face limitations associated with their sensitivity, turnaround time, cost, and/or accessibility, which hinder their effectiveness in real-world settings. Electrochemical biosensors have recently gained attention as innovative diagnostic tools because they deliver highly sensitive and specific results quickly, making them ideal for point-of-care testing. Incorporating three-dimensional (3D) structured materials can enhance biosensor performance by expanding the binding surface area for biorecognition probes and optimizing signal transduction mechanisms. This review highlights the current understanding of influenza viruses and presents the latest developments in electrochemical biosensing technologies. We emphasize the integration of materials such as metal nanoparticles, carbon-based materials, and metal–organic and covalent–organic framework-based materials that can provide 3D surfaces. These strategies enable the sensitive and selective detection of multiple influenza strains. The development of 3D probe immobilization technologies and biosensor engineering has shown promise for practical clinical implementation and large-scale diagnostic use, potentially contributing to improved influenza surveillance and public health outcomes.

## Abstract 2

3D capture probe immobilization on nanostructured electrochemical biosensors allows sensitive, selective influenza detection, enabling rapid, low-cost, point-of-care diagnostics for effective infection control.

## Graphical abstract

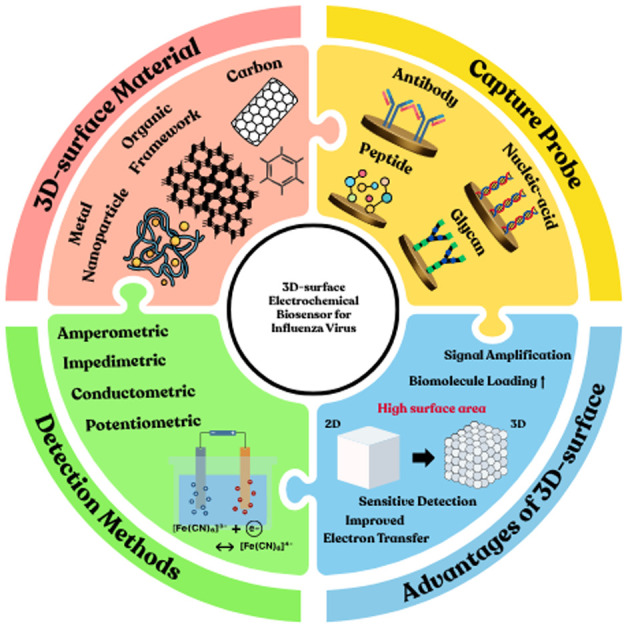

## 1 Introduction

Influenza, an acute respiratory infection caused by influenza viruses, is responsible for over 3 million severe cases and 650 000 respiratory disease-related deaths globally each year. Influenza viruses are categorized into four types: A, B, C, and D.^1^ Of these, influenza A and B primarily cause seasonal epidemics during winter, with only influenza A capable of triggering pandemic outbreaks.^2^ High-risk groups such as children, the elderly, and individuals with chronic conditions are at greater risk of severe complications and mortality from influenza.^3,4^ For example, in 2018, an estimated 109.5 million influenza episodes, 0.9 million hospital admissions, and up to 34 800 deaths occurred globally among children under 5 years of age.^5^ The hospitalization rates and complication frequencies for infants and children under two years old are also comparable to those of high-risk adults and elderly individuals,^3,6^ while pregnant women are at higher risk of severe illness and preterm birth due to changes in their immune and respiratory functions.^7–9^ These statistics highlight the seriousness of influenza infections and the urgent need for rapid and accurate diagnostic methods.

Early diagnosis of influenza allows for timely antiviral treatment and preventive measures, reducing the duration and severity of its symptoms and preventing the spread of the disease. Various influenza diagnostic methods are currently in use,^10^ each with particular advantages and limitations in terms of time, sensitivity, specificity, and cost.^11^ For example, traditional virus culture methods offer high specificity but take several days to produce results and require specialized laboratory equipment,^12^ while rapid antigen tests provide quick results but may have lower sensitivity for the detection of specific virus strains.^13^ In contrast, polymerase chain reaction (PCR) tests ensure high sensitivity and specificity but are costly and require complex equipment and skilled personnel. Therefore, new technologies that offer rapid but highly sensitive and specific results at a low cost are required. These technologies also need to be suitable for point-of-care (POC) testing and large-scale screening to enhance efficiency.

Electrochemical biosensors have emerged as a promising strategy to meet these requirements.^14^ They convert biological interactions into measurable electrical signals, providing high sensitivity, specificity, and fast response times.^15,16^ They can also be miniaturized and integrated into portable devices, making them suitable for POC applications.^17,18^ Recent advances in nanotechnology and materials science have enhanced the performance of electrochemical biosensors, allowing for the detection of low concentrations of virus particles.^19^

Technologies based on the immobilization of capture probes on three-dimensional (3D) surfaces has increasingly gained attention.^20^ This is because 3D surfaces provide more binding sites than traditional two-dimensional (2D) surface coatings, enhancing the sensitivity and specificity.^21^ Various probes have been used in biosensor technologies, including oligonucleotides,^22,23^ antibodies,^24^ peptides,^24–27^ and glycans,^28^ each of which offers distinct advantages in terms of binding efficiency and signal amplification. These probes significantly enhance sensor performance when immobilized on advanced 3D structures such as graphene, hydrogel, and porous silica. For example, 3D graphene oxide structures have been shown to improve electrochemical performance by facilitating electron transfer, while hydrogels provide an ideal biocompatible matrix for the capture of biomolecules.^29,30^

Several surface modification techniques have been developed to produce advanced 3D coatings, including spin coating,^31^ dip coating, electrodeposition,^32^ and layer-by-layer assembly.^33^ Spin coating forms uniform thin layers, which are useful for sensors that require high sensitivity, while dip coating offers a straightforward method for depositing materials onto 3D surfaces. Electrodeposition is often employed to precisely place conductive materials such as gold nanoparticles (AuNPs) on 3D scaffolds. Additionally, layer-by-layer assembly is used for the controlled fabrication of multi-layered structures, enhancing the functionality and specificity of the resulting biosensor. These surface modification techniques combined with innovative 3D materials can be used to produce biosensors with enhanced sensitivity, specificity, and stability for next-generation diagnostic platforms.

**Scheme 1:**
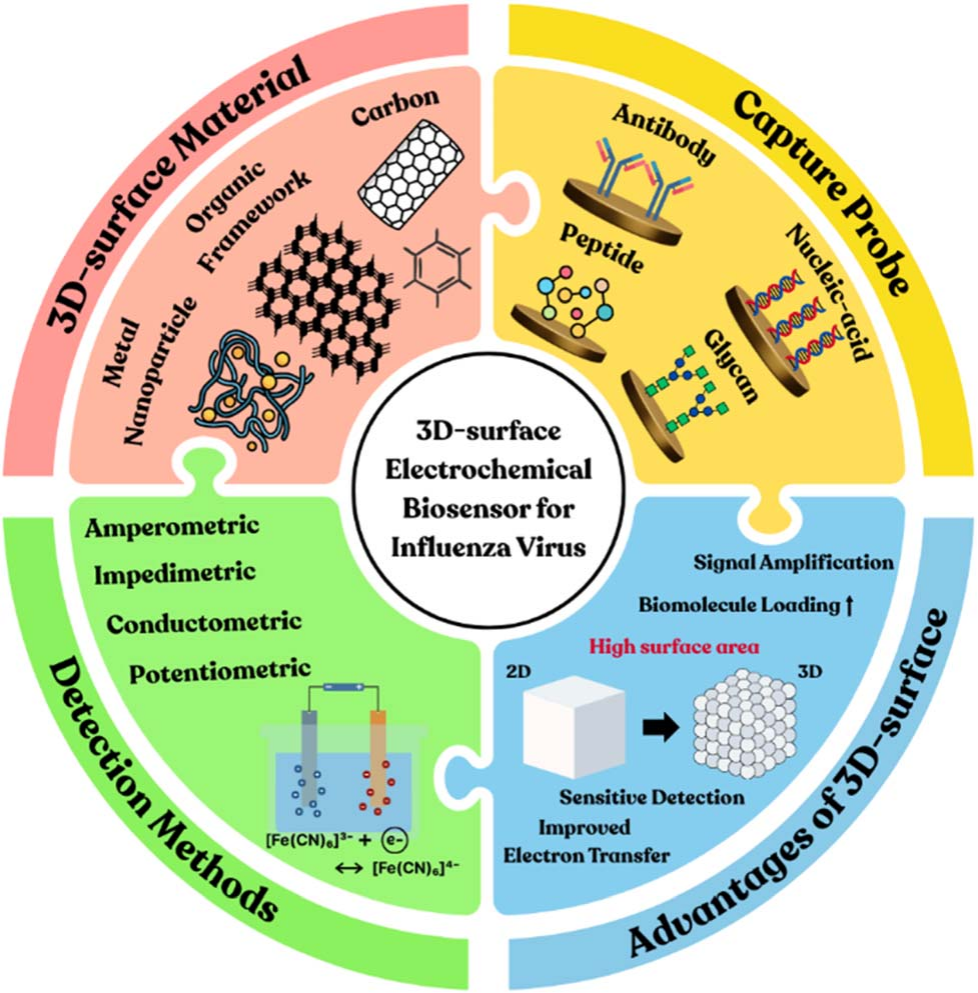
Overview of 3D surface-based electrochemical biosensors for influenza virus detection: classification by sensor type, capture probes, and surface materials.

This review focuses on the development and application of electrochemical biosensors with capture probes immobilized on their 3D surface for influenza virus detection, emphasizing their potential to overcome the limitations of existing diagnostic methods. As outlined in Scheme 1, this work aims to provide insights into future directions for influenza virus detection technologies.

## 2 Viruses and influenza

### 2.1 Structure and characteristics of viruses

Viruses are submicroscopic infectious particles that are much smaller than bacteria and can only replicate within living host cells.^34^ They lie at the boundary between living and non-living things, thus they are often referred to as acellular biological entities. The structure of a virus primarily consists of two main components: genetic material (either DNA or RNA) and a protein shell called a capsid.^34^ Viruses use their nucleic acids to transmit and replicate their genetic information within host cells. The capsid, which is composed of multiple small subunits known as capsomers,^34^ protects the nucleic acids and provides structural support when invading the host cell. The capsid has a regular symmetrical shape, such as an icosahedron that is nearly spherical or helical.^34^ Some viruses also have a lipid envelope surrounding their capsid that is derived from the host cell's membrane. Many enveloped viruses have spike proteins on their surface that attach to host cells, allowing them to infect a wide range of organisms, including animals, plants, and microorganisms.^35^

RNA is the genetic material of influenza viruses. Their genome consists of 7 or 8 segmented negative-sense RNA strands, with each RNA segment containing 1 or 2 genes.^35,36^ This segmented genome structure facilitates genetic recombination. Influenza viruses do not have RNA integrity enzymes, which allows these viruses to mutate very rapidly.^37^ These mutations can create new antigen types that evade recognition by the immune system. In particular, antigenic drift and antigenic shift are commonly observed in influenza viruses, contributing to new pandemics. Influenza viruses also have an envelope containing hemagglutinin (HA) and neuraminidase (NA) proteins. These surface antigens determine the infectivity and transmissibility of the virus.^36^ HA proteins help virus particles attach to the respiratory cells of the host by binding to sialic acid, which is the first step for the virus to enter the cell.^35^ NA proteins break down sialic acid on the host cell surface, facilitating the spread of newly formed virus particles to surrounding cells.^38^

In biosensors, the surface antigens of influenza viruses not only exhibit significant antigenic variation but also undergo extensive glycosylation, which affects the accessibility and binding efficiency of probes. HA forms a trimeric spike, where the HA1 subunit constitutes a globular head domain responsible for receptor binding and major antigenic epitopes.^39^ In contrast, the HA2 subunit forms a more conserved and structurally buried stalk domain.^39^ NA adopts a mushroom-shaped tetrameric structure with a stalk of variable length that elevates its enzymatic head above the viral envelope.^40^ These structural features govern the physical accessibility of capture probes. The HA head extends roughly 13–14 nm above the viral membrane, providing numerous potential binding sites for antibodies or aptamers. In contrast, the orientation and stalk length of NA influence the spatial accessibility of its active site. The high density and multivalency of HA spikes amplify signal responses in biosensors, significantly enhancing electrochemical signals, particularly in impedance- or current-based electrochemical platforms.^41,42^ However, this accessibility is strongly regulated by N-linked glycosylation, where dynamic glycan patterns can conceal specific epitopes, thereby interfering with interactions between the viral target and immobilized probes such as antibodies or aptamers, causing steric hindrance and reducing sensitivity.^43^ Moreover, continuous antigenic drift and reassortment of influenza A viruses increase the risk of cross-reactivity and reduce sensor specificity.^44^ Overall, viral structural characteristics, including surface proteins, glycosylation, and antigenic variability, have a significant impact on biosensor sensitivity and specificity. Achieving a low limit of detection (LOD) requires efficient capture of viral surface antigens or genetic material. Therefore, comprehensive consideration of these viral structural elements during target selection and sensor design is essential for developing robust biosensor platforms capable of sensitive and broad-range influenza virus detection.^45^ Because of the variability and rapid transmission of influenza viruses, they cause millions of infections and many deaths worldwide each year, thus they need to be carefully monitored.^37^

### 2.2 Types of influenza viruses

Influenza viruses are classified into four main types—A, B, C, and D—that differ in their transmissibility and pathogenicity.^46,47^

Type A influenza viruses are the most widely spread, common, and severe type.^46^ They are highly contagious and can infect humans and other animals, such as pigs and birds, often leading to large-scale pandemics.^37^ Type A viruses are further divided into subtypes based on the two main surface antigens, HA and NA, with H1N1 and H3N2 being representative examples.^36^ The high antigenic variability of type A—driven by frequent antigenic drift and occasional antigenic shift—complicates the design of electrochemical biosensors, since single-probe systems may fail to recognize newly emerging subtypes. To achieve robust detection, multiplexed panels or probes targeting conserved proteins such as matrix protein M1 are often employed, and surface modification strategies (*e.g.*, nanomaterials) are applied to enhance probe density and binding despite glycosylation-induced steric hindrance.^48,49^ New variants can evade the immune system through antigenic drift and antigenic shift, contributing to outbreaks and leading to severe respiratory diseases and serious complications such as pneumonia.

Type B influenza viruses are less transmissible and less severe than type A.^37^ These viruses only infect humans and are not transmitted between animals.^50^ While type B viruses can cause pandemics, they are less common than type A. Type B is not divided into subtypes but is classified into two lineages: Victoria and Yamagata.^50^ For biosensor design, both lineages must be considered to avoid false negatives, as even minor antigenic drift can reduce probe binding affinity. Nevertheless, probe updates are required less frequently for type B than for type A due to the slower evolution of B-lineage viruses.^51,52^ These lineages cause regional outbreaks and exhibit less genetic variability compared to type A viruses. Consequently, vaccines tend to remain effective for longer, although the risk of severe infections for vulnerable populations remains.^50^

Type C influenza viruses provoke the mildest symptoms.^37,53^ They primarily cause mild respiratory illnesses similar to colds and can infect humans and animals; however, the likelihood of large-scale outbreaks is much lower than for type A and B viruses. Given their low antigenic variability and minimal clinical relevance, type C viruses are rarely targeted in electrochemical biosensor development, as detection priorities focus on types A and B.^51,52^ Most of the population is already immune, resulting in few serious diseases. Therefore, this group of influenza viruses is not a target for vaccine development. Type D influenza viruses primarily infect livestock such as cattle and pigs, and to date, there have been no reported cases of human infection.^46,53^ Similar to type C, the absence of significant human transmission and low antigenic variability makes type D of limited relevance to biosensor applications.^51,52^

### 2.3 Detection methods for influenza viruses

Various traditional and advanced methods for the detection of influenza viruses have been developed, each with its own advantages and disadvantages. Traditional techniques include virus cultures and serological tests. Viral culture tests cultivate specimens to confirm the presence of a viral infection^37^ and, though this method can be accurate, it requires 48–72 h to obtain results, making rapid confirmation challenging. This test also requires a laboratory environment and specialized equipment^43^ and is thus primarily utilized to confirm infections during large-scale outbreaks. Serological tests measure the levels of antibodies for the influenza virus in blood samples,^54^ proving information on the severity of the disease or stage of infection. This is because studies have shown that antibody levels, such as IgM and IgG, may differ between the phases of infection and can correlate with the severity of symptoms. This method is generally helpful in determining recent infections and evaluating immunity to influenza but requires considerable analysis time.^37^

PCR is a standard method that directly detects viral RNA, offering high sensitivity and specificity even for novel influenza viruses. However, it requires expensive equipment and long analysis times.^1,54^ Reverse transcription (RT)-PCR is an improved version of PCR that provides faster and more accurate results and has the advantage of being able to differentiate between virus subtypes.^36,54,55^ In addition, next-generation sequencing provides high accuracy and detailed genetic information through a complete analysis of the viral genome,^37^ facilitating the tracking and monitoring of new viral variants. Nevertheless, it also has a long analysis time and higher costs, making it less suitable for real-time diagnostics.^56^

Enzyme-linked immunosorbent assays (ELISA) use antigen–antibody reactions to detect viral antigens or antibodies induced by viruses.^37^ While this approach can analyze large volumes of samples simultaneously, it has relatively low sensitivity.^1^ Immunofluorescence is another method that uses antibodies labeled with fluorescent materials to detect viral antigens in real time, producing rapid results and providing morphological information about viral particles.^1,37^ However, this method requires expensive fluorescent microscopy and may have lower sensitivity for specimens with low viral loads.^54,56^ In addition to ELISA and traditional immunofluorescence, fluorescence-linked immunosorbent assays (FLISA) and fluorescent enzyme-linked immunosorbent assays (FELISA) have been developed to enhance the sensitivity and specificity. These methods allow for faster readouts and the more precise detection of low viral loads compared to traditional ELISA, making them suitable for clinical diagnostics. FELISA combines the amplification capability of enzyme reactions with fluorescence detection, improving the signal clarity and sensitivity, which is particularly useful with low viral concentrations. This method can produce results with less background noise, making it a valuable tool for accurate diagnosis, even though it is more costly and requires specialized equipment.

Biosensors represent a ground-breaking approach to influenza detection, offering innovative methods for infectious disease surveillance and diagnostics with rapid and accurate results. These devices are categorized based on their detection mechanisms, including optical, fluorescence-based, and electrochemical biosensors. Fluorescence-based biosensors offer high sensitivity and specificity, producing accurate results even at low viral loads. Other biosensors, such as rapid antigen detection tests and CRISPR-based diagnostics, are effective for on-site detection. Rapid antigen detection tests are user-friendly tools that detect specific antigens on the surface of a virus, providing results within 5 to 10 min and making them suitable for POC testing.^54,57^ CRISPR-based diagnostics can accurately target viral RNA or DNA, identifying influenza types A and B and their subtypes within 15 min.^58,59^ Combining these diagnostic techniques can significantly enhance the ability to detect influenza and other viruses.^54^ However, their sensitivity may be insufficient for detecting low viral loads.

Electrochemical biosensors, in contrast, offer significant advantages, including higher sensitivity, rapid response times, and the precise detection of low concentrations of viral particles. These attributes make electrochemical biosensors particularly valuable in clinical diagnostics, where early and accurate detection is essential. The following review provides an in-depth exploration of the features and benefits of electrochemical biosensors, highlighting how they enhance and complement existing diagnostic methods.

## 3 Electrochemical biosensors

### 3.1 Principles and applications of electrochemical biosensors

Electrochemical biosensors are particularly effective for the early diagnosis of infectious diseases because of their high sensitivity and rapid response time, making them invaluable in medical diagnostics.^59,60^ Electrochemical biosensors can detect specific analytes by measuring changes in the current, voltage, or impedance caused by molecular interactions.^61–63^

One of the key advantages of electrochemical biosensors is their ability to provide real-time results, allowing for the immediate evaluation of a patient's condition, facilitating early intervention and treatment. These sensors are also easy to mass-produce at low cost.^63^ The compact size, portability, and affordability of electrochemical biosensors make them ideal for POC testing, where rapid decision-making is essential.^59^ Due to these advantages, electrochemical biosensors have been widely used in multiple fields, including detecting pathogens in blood, saliva, and other biological samples,^62^ environmental monitoring (*e.g.*, pollutants and heavy metals),^64^ and food safety testing (*e.g.*, allergens and bacterial contamination).^65^ These sensors are also easily integrated into wearable or implantable devices for continuous health monitoring.^62,66^ However, electrochemical biosensors are associated with limited signal reproducibility and external interference from non-target molecules.^67,68^ Recent developments in electrochemical biosensors have focused on overcoming these limitations by incorporating nanotechnology, artificial intelligence, and 3D coating materials.^59^ Multiplexed electrochemical biosensors, which enable the simultaneous detection of multiple analytes, have gained significant attention due to their ability to monitor a broad range of biomarkers in real-time. This multiplexing capability is especially valuable in digital health systems where continuous data collection, real-time analysis, and remote monitoring are essential for disease management and patient care.^62,69^ With the integration of multiplexed biosensors, multiple conditions can be diagnosed simultaneously, significantly improving the efficiency of diagnostics. Furthermore, the use of 3D coating materials has improved sensor performance by increasing the surface area of electrodes, boosting signal sensitivity, and enabling the detection of low-concentration targets with greater accuracy.^59,67,70^

### 3.2 Types of electrochemical biosensor

**Fig. 1:**
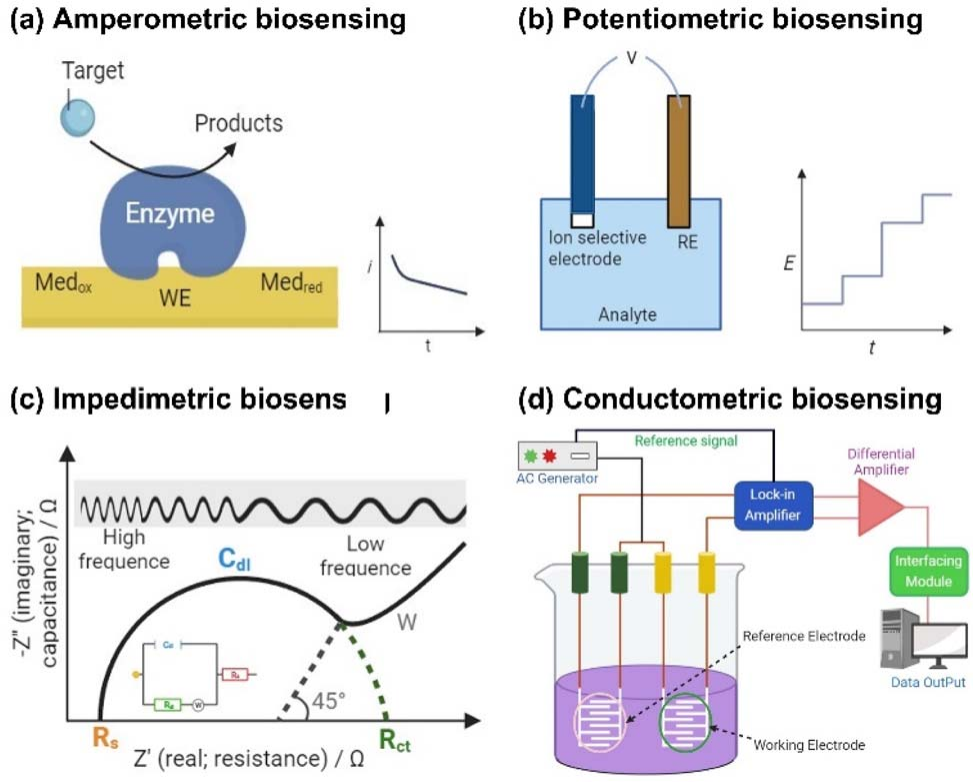
Representative diagrams of four types of electrochemical biosensors: (a) amperometric, (b) potentiometric, (c) impedimetric, and (d) conductometric.

Electrochemical biosensors can be classified into four main types based on their operating principles and detection mechanisms: amperometric, potentiometric, impedimetric, and conductometric (Fig. 1). Each type differs in the method they use to transduce biochemical signals into measurable electrical outputs, offering specific advantages and the ability to tailor the choice of biosensor to specific applications to detect and analyze a wide range of biomolecules. A comparative summary of these major electrochemical biosensor types, highlighting their advantages, limitations, and typical applications, is provided in Table 1.

**Table 1:** Comparative summary of major electrochemical biosensor types

Type	Principle	Advantages	Limitations	Application
Amperometric	Detects current generated from redox reactions; the current is directly proportional to analyte concentration	High sensitivity with broad detection range; rapid response enabling real-time monitoring; simple design and easily miniaturized	Susceptible to interference from other redox-active species; requires redox mediators or enzymatic labels	Quantification of viral RNA or antigens using enzymatic or redox-based labeling strategies
Potentiometric	Monitors changes in electrochemical potential (under near-zero current) using ion-selective electrodes, based on the Nernst equation	Minimal power and sample consumption; non-destructive measurement; simple device	Limited sensitivity for non-ionic analytes; prone to ion interference; slow equilibrium response	pH measurement, ion concentration analysis (Na^+^, K^+^, Ca^2+^), and gas sensing (NH_3_, CO_2_)
Impedimetric	Evaluates changes in impedance at the electrode surface following analyte bindin	Label-free detection; highly sensitive to interfacial changes; supports real-time analysis	Complex instrumentation and data interpretation; strongly influenced by electrode surface quality	Detection of DNA/RNA, protein–protein interactions, and cell adhesion monitoring
Conductometric	Measures variations in conductivity caused by changes in ion concentration	Simple and low-cost; no counter electrode required; easily miniaturized	High susceptibility to temperature and ionic strength fluctuations; poor selectivity in complex matrices	Enzyme-based assays, salinity detection, and water quality monitoring

#### 3.2.1 Amperometric biosensors

Amperometric biosensors are among the most widely studied electrochemical biosensors. A typical amperometric biosensor consists of three key components: a working electrode (usually made of gold, platinum, or carbon), a reference electrode, and a counter electrode. The fundamental electrochemical principle governing amperometric biosensors is Faraday's law, which states that the amount of material oxidized or reduced at the electrode is directly proportional to the charge transferred. The Cottrell equation describes the current of an amperometric sensor:
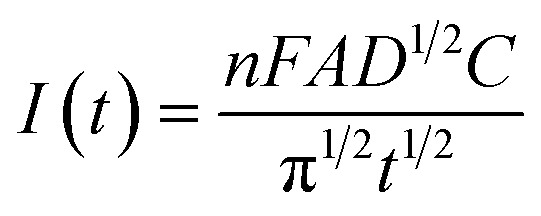
where *n* represents the number of electrons involved in the redox reaction, *F* is the Faraday constant, *A* is the electrode surface area, *D* is the diffusion coefficient of the analyte, *C* is the analyte's bulk concentration, and *t* indicates time.^71,72^ This equation delineates the relationship between the current and analyte concentration, enabling precise quantification. Due to their high sensitivity and specificity, amperometric biosensors are extensively used in clinical diagnostics, environmental monitoring, and food safety applications.^71^

#### 3.2.2 Potentiometric biosensors

Potentiometric biosensors are employed to detect various analytes, especially when changes in ion concentration and activity occur. These sensors utilize ion-selective electrodes to measure the difference in potential between the working electrode and reference electrode, with the resulting potential directly correlated to the analyte concentration.^73–75^ The fundamental principle is described by the Nernst equation, which defines the relationship between the electrode potential and the ion concentration in a sample. The Nernst equation is expressed as follows:
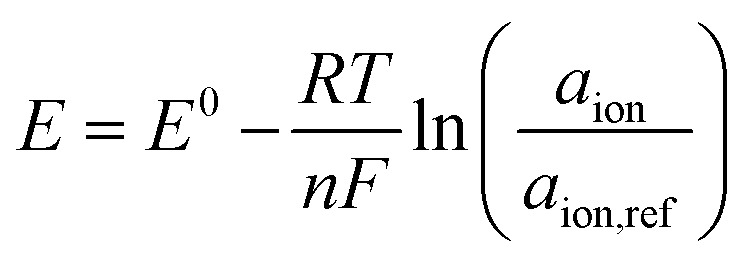
where *E* is the measured electrode potential, *E*^0^ is the reference potential, *R* is the gas constant, *T* is the absolute temperature, *n* is the number of electrons involved in the reaction, *F* is the Faraday constant, *a*_ion_ is the activity of the ions in the sample, and *a*_ion, ref_ is the activity of the ions in the reference solution.^76^

#### 3.2.3 Impedimetric biosensors

Impedimetric biosensors can detect biomolecular interactions by measuring the impedance or resistance to an alternating current at the electrode–electrolyte interface.^77,78^ Impedance (*Z*) is a complex parameter that includes resistance, capacitance, and inductance. The total impedance can be expressed as
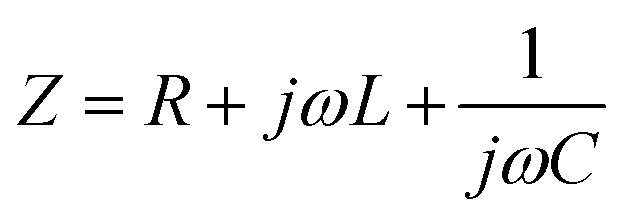
where *R* represents the resistive component, *L* is the inductive component, *C* is the capacitive component, *ω* is the angular frequency of the applied AC signal, and *j* is an imaginary unit. In most biosensing applications, the inductive component is often negligible.

When biomolecules such as proteins, DNA, or pathogens bind to the electrode surface, they modify the charge distribution, influencing the impedance at the interface. These changes are generally quantified by measuring variation in the charge transfer resistance (*R*_ct_) and double-layer capacitance (*C*_dl_) through electrochemical impedance spectroscopy (EIS). When antigens (or pathogens) bind to the surface, *C*_dl_ decreases, *R*_ct_ increases, and the solution resistance may vary. Changes in *R*_ct_ can be used to evaluate the presence and concentration of pathogens. A low *R*_ct_ indicates efficient electron transfer at the electrode surface, implying the absence of pathogens. Conversely, a high *R*_ct_ arises when antigen–antibody binding hinders electron movement, confirming the presence of pathogens.^79^

#### 3.2.4 Conductometric biosensors

Conductometric biosensors operate by detecting changes in the electrical conductivity of a solution due to biochemical reactions that affect the concentration or mobility of charged species. These sensors are especially valuable for identifying analytes that strongly influence the ionic strength, such as enzyme–substrate interactions. In addition to their ability to detect these changes, conductometric biosensors offer the advantage of not requiring a reference electrode, which can reduce equipment and analysis costs.^80^ The fundamental principle of these biosensors is based on the relationship between conductivity (*σ*) and ion concentration in solution, which is expressed as follows:
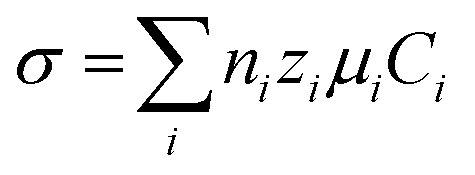
where *σ* is the conductivity, *n*_*i*_ is the number of ions of species *i*, *z*_*i*_ is the charge number of the ions, *μ*_*i*_ is the ion mobility, and *C*_*i*_ is the ion concentration.^81,82^ When the target analyte interacts with the sensor, a biochemical reaction occurs, resulting in the generation or consumption of charged species, such as protons, hydroxide ions, or metal cations, which alters the conductivity of the solution.

Amperometric, potentiometric, impedimetric, and conductometric biosensors have distinct advantages but are all valuable tools in clinical diagnostics, environmental monitoring, and food safety. However, these electrochemical biosensors face a number of issues that affect their performance and reliability. One major concern is their poor selectivity, which can lead to inaccurate results due to cross-reactivity with nontarget molecules and matrix effects in complex biological samples. Another issue is their poor stability because these biosensors can degrade over time due to contamination by proteins, salts, or other substances. This can be alleviated by modifying the sensor surface with biocompatible coatings and protective layers to prevent contamination and extend the sensor's operating life. Nanotechnology has also played a key role in overcoming these limitations by increasing the sensor's surface area through nanostructures and utilizing signal amplification methods such as enzymatic reactions or fluorescent tags, thus significantly improving detection performance. Recent advances in nanotechnology have enabled the development of small, high-performance biosensors suitable for portable and wearable applications.

## 4 Electrochemical biosensors for influenza detection

Electrochemical biosensors have emerged as promising tools for viral diagnostics, particularly in the detection of influenza, due to their sensitivity, potential for miniaturization, and compatibility with portable formats. Of the various factors affecting sensor performance, the structure of the electrode surface is particularly important, influencing probe immobilization, electron transfer kinetics, and the detection sensitivity. This section explores recent advances in electrode surface engineering, with a focus on two main design strategies: 2D planar surfaces and 3D structured architectures. Section 4.1 provides an overview of conventional and emerging 2D surface-based biosensing platforms, while Section 4.2 focuses on recent advancements incorporating 3D nanostructures and hybrid materials.

### 4.1 2D surface-based electrochemical biosensors

**Fig. 2:**
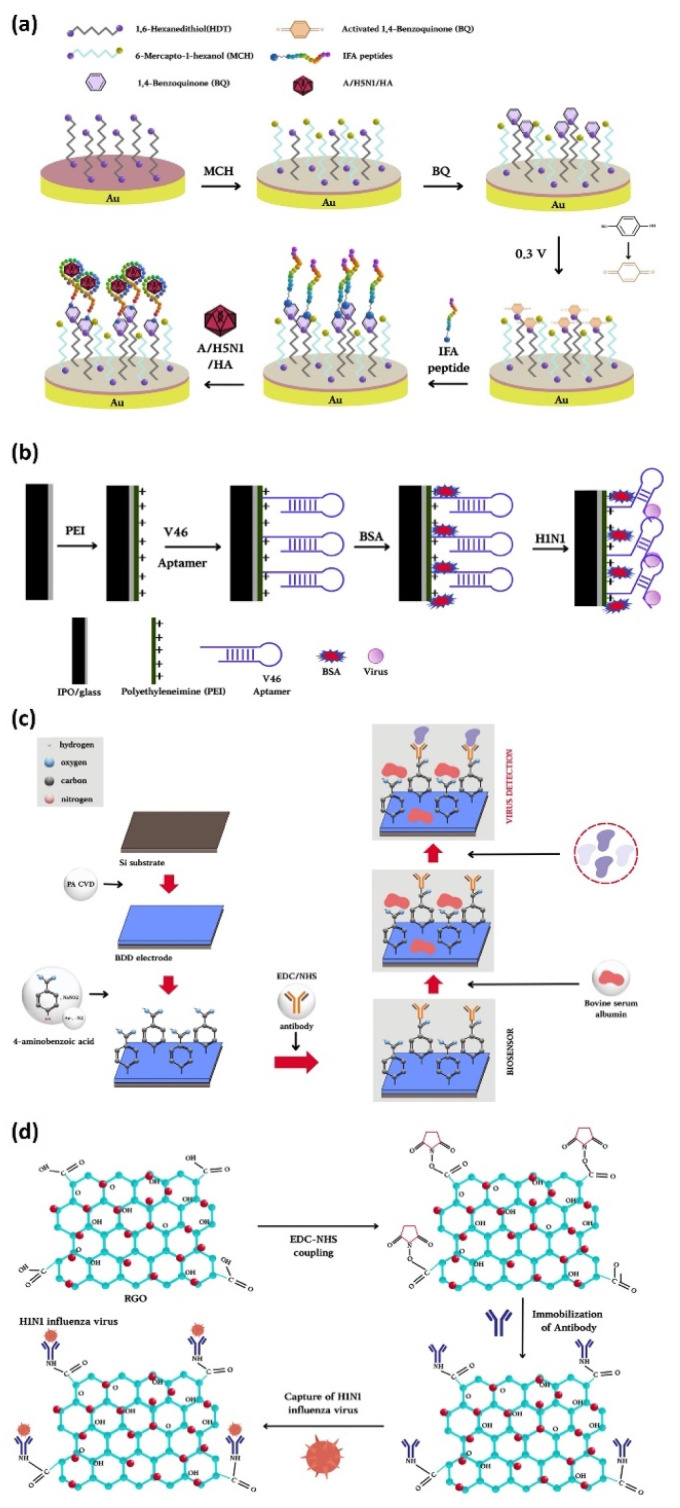
Schematic representations of typical 2D electrode configurations and surface structures used in electrochemical biosensors: (a) a gold (Au) electrode with self-assembled monolayers (SAMs) facilitating biomolecule immobilization, (b) an indium tin oxide (ITO) electrode offering transparent conductivity for combined optical–electrochemical applications, (c) a boron-doped diamond (BDD) electrode characterized by a wide potential window, low background current, and superior chemical stability, and (d) a reduced graphene oxide (rGO) electrode featuring a large surface area and abundant functional groups to promote electron transfer and enhance probe immobilization. These illustrations emphasize the structural and functional characteristics of each electrode type described in Section 4.1.

The surface architecture of electrodes is one of the key determinants influencing the performance of electrochemical biosensors. In particular, 2D platforms, such as planar surfaces and nanomaterials, offer facile fabrication, chemical stability, and compatibility with a wide range of surface modification strategies.^83,84^ They have thus been widely utilized in biosensor development to create reproducible and versatile interfaces suitable for the immobilization of capture probes and the detection of viral targets. Nevertheless, 2D systems face practical limitations, including restacking, restricted ion diffusion, and finding a balance between surface area and electrical conductivity. These factors restrict the effective surface area and mass transport, reducing sensitivity to low-abundance biological targets in biosensing platforms.^85^ To overcome these limitations, extensive research has focused on optimizing electrode materials and surface modification strategies (Fig. 2).

Gold (Au) and indium tin oxide (ITO)-based electrodes have become standard platforms for electrochemical biosensor research due to their excellent electrochemical stability and compatibility with a wide range of surface functionalization strategies, making them particularly suitable for stable biomolecule immobilization. Au-based electrodes, which are available in various configurations such as bulk Au,^86^ Au disk electrodes (GDEs),^87^ and screen-printed Au electrodes (SPGEs),^88^ have been widely employed in electrochemical biosensors due to their superior biocompatibility and ease of surface functionalization. They have a strong affinity for thiol groups, which leads to the formation of self-assembled monolayers (SAMs) for the efficient and stable immobilization of biomolecular probes. For example, SAMs formed using 1,6-hexanedithiol or cysteine derivatives have been employed to immobilize peptides, DNA probes, and other recognition elements targeting influenza virus antigens, including hemagglutinin.^88^ These surface modification strategies facilitate rapid and reproducible probe attachment, ultimately enhancing the sensitivity and specificity of influenza detection platforms. ITO is a well-known electrochemically active transparent electrode that is typically deposited on glass substrates. Due to its flat surface and high conductivity, it is commonly employed in biosensors. Notably, aptamer-functionalized ITO glass electrodes have been reportedly used for the discrimination and detection of various influenza A virus subtypes, including H1N1 and H3N2.^89^

Various novel materials have also been investigated to enhance the sensitivity and selectivity of electrochemical biosensors. Of these, boron-doped diamond (BDD) electrodes have attracted significant attention for the design of highly selective and sensitive immunosensors. BDD exhibits a wide potential window, low background current, excellent chemical stability, and biocompatibility, making it a key material for next-generation electrochemical biosensors. These characteristics enable the precise measurement of weak biological signals with minimal interference, thus overcoming the limitations of traditional metallic electrodes. In previous research, polyclonal antibodies targeting the matrix protein 1 (M1) of the influenza virus were covalently immobilized onto a BDD electrode, resulting in a biosensor capable of rapid and ultrasensitive detection within 5 min, with a detection limit as low as 1 fg mL^−1^. Functionalized BDD surfaces have been shown to facilitate electron tunneling and offer superior charge transfer, enhancing the efficiency of electrochemical reactions.^47^ Consequently, the electrical performance of BDD is stronger than that of Au electrodes.

An advanced version of BDD is nanocrystalline boron-doped diamond (B:NCD), which has a fine-grained crystal structure that significantly increases the active surface area and enhances electron mobility and surface reactivity. B:NCD films have been fabricated on p-type silicon substrates *via* spin coating using a dispersion of detonation nanodiamond (DND) in dimethyl sulfoxide (DMSO) with polyvinyl alcohol (PVA), followed by the immobilization of M1-specific antibodies to develop a highly sensitive impedance-based biosensor. The smaller B:NCD grain size increases the sp^2^ carbon contribution and quantum confinement effects, improving its sensitivity. Moreover, the hydrogen-terminated surface of B:NCD enhances its hydrophobicity, minimizing nonspecific protein adsorption and preserving protein integrity. These characteristics suggest that B:NCD may outperform conventional BDD in biosensor applications.^90^

Another prominent strategy for overcoming the limitations of traditional metallic electrodes involves decorating the electrode surface with 2D nanomaterials. In particular, reduced graphene oxide (rGO) has emerged as a promising electrode material due to its large specific surface area, high conductivity, and abundant oxygen-containing functional groups. In a previous study, a Au electrode patterned *via* photolithography on a glass substrate was modified with a cystamine-based SAM, followed by dip-coating in rGO nanosheets. The carboxyl groups on the rGO surface were involved in EDC/NHS coupling with antibodies, leading to stable immobilization without additional linkers. Structural analysis revealed that the rGO sheets formed wrinkled and layered flakes (approximately 6–8 layers thick), increasing the effective surface area and roughness and promoting electron transfer at the electrode/electrolyte interface. AFM analysis revealed a surface roughness of approximately 45.4 nm, facilitating the effective adsorption of antibodies and viral particles and enhancing sensor sensitivity. Furthermore, the curled or folded edges observed under electron microscopy contributed to the mechanical stability and improved charge mobility. The resulting rGO-modified electrodes exhibited a redox peak current up to 8.5 μA, significantly higher than electrodes modified with cystamine alone, which was attributable to the excellent conductivity and unique electronic properties of rGO. The oxygen-rich functional groups on the rGO surface, such as carboxyl groups, enhanced its hydrophilicity and dispersion in aqueous media, contributing to improved sensor performance. The rGO-based sensor achieved a detection limit of 0.5 PFU per mL for H1N1 influenza and a linear response over the 1–10^4^ PFU per mL range (*R*^2^ = 0.99).^91^

However, chemically reduced rGO is typically prepared using strong oxidants and reductants and thus suffers from residual toxicity and structural defects, leading to compromised conductivity and biocompatibility. To address this issue, a thermally reduced graphene oxide (TrGO) has been developed using the natural biopolymer shellac, a biocompatible and biodegradable resin secreted by female lac insects, as a precursor. The shellac was pyrolyzed at 700 °C in an inert atmosphere to yield a highly crystalline TrGO film. TrGO flakes were then drop-cast onto an ITO/glass substrate to produce the electrode. The resulting TrGO exhibited high crystallinity and low sheet resistance, significantly improving the electrical conductivity. The multilayered graphene sheets (approximately 18 nm thick) contributed to a more ordered structure, enhancing the stability and reproducibility of the sensor. Electrochemical evaluation found that the TrGO/ITO electrodes generated substantially higher redox peak currents than GO or hydrazine-reduced rGO. EIS and differential pulse voltammetry (DPV) analysis corroborated the enhanced charge transfer capabilities of the TrGO-based electrodes. Additionally, the phenolic-OH groups on TrGO surface enabled covalent antibody immobilization without further chemical treatment, providing a practical and stable platform for biosensor construction. The multilayered, wrinkled structure of TrGO also enhanced the effective surface area, improving both antibody loading and analyte accessibility, thus maximizing the sensor sensitivity.^92^

### 4.2 3D surface-based electrochemical biosensors

While 2D surface-based electrochemical biosensors have undergone various modifications to improve their sensitivity and selectivity, they still face inherent limitations due to their low surface area and restricted probe immobilization capabilities. To overcome these issues, 3D surface-based electrochemical biosensors have been actively explored. Incorporating 3D architectures significantly enhances the effective surface area, allowing for a higher density of immobilized bioreceptors and facilitating more efficient electron transfer pathways, collectively contributing to stronger electrochemical signals. The porous and hierarchical nature of 3D materials also promotes molecular diffusion and accelerates the reaction kinetics. In this section, we examine 3D surface-based electrochemical biosensors for influenza virus detection developed using (i) metal NPs, (ii) carbon-based materials, and (iii) organic frameworks as the functionalized materials. These approaches offer unique advantages regarding conductivity, surface functionality, and integration with detection platforms, although some limitations in terms of reproducibility, stability, or cost may remain. Tables 2–4 summarize representative studies according to the functionalized material involved and provide a comparative overview of the structural characteristics and performance metrics of the resulting sensor system.

**Table 2:** A comparison of metal nanoparticles-based electrochemical biosensors for influenza virus detection

Electrodes^a^	3D surface materials^b^	3D surface materials immobilization method	Capture probes	Capture probe immobilization method	Analytes (influenza subtypes)	Detection methods^c^	Limit of detection	Detection range	Reference
Carbon	AuNPs	Physical adsorption	Antibody	Chemical conjugation (glutaraldehyde)	H1N1	CV, DPV	0.25 pg mL^−1^	0.25–5 pg mL^−1^	24
Au disk	AuNPs	*In situ* synthesis	DNA	Thiol–Au reaction	Influenza B	DPV	86.4 amol L^−1^	1.0 fmol L^−1^–1.0 nmol L^−1^	95
PGE	AuNPs	Physical adsorption	DNA	Thol–Au reaction	Influenza B	CV, DPV	54 pM	—	96
Au	ZnO nanorods	*In situ* synthesis	Antibody	Electrostatic interaction	H1N1, H5N1, H7N9	Amperometry (*I*–*t* curve)	1 pg mL^−1^	1 pg mL^−1^–10 ng mL^−1^	97
Au	TiO_2_–adenine nanocomposites	Adsorption *via* Adenine–Au affinity	DNA	EDC/NHS reaction; van der Waals interaction	H1N1	CV, EIS, DPV	6.7 × 10^−8^ ng mL^−1^	3 × 10^−8^–3 × 10^−3^ ng mL^−1^	98
SPGE	TiO_2_–guanine nanocomposites	Adsorption *via* Guanine–Au affinity	DNA	Van der Waals interaction	H1N1	CV, EIS	0.00024 ng/6 μL	0.0002–20 ng/6 μL	99
Au	Porous AuNPs	Physical adsorption; electrostatic interaction	DNA	Thiol–Au reaction	H5N1	CV	1 pM	1 pM–1 μM	100
Carbon	*Trans*-dimensional NCGF	*In situ* synthesis	Antibody	EDC/NHS reaction	Influenza A virus	DPV	13.14 fg mL^−1^	100 fg mL^−1^–10 ng mL^−1^	101

a PGE, printed gold electrode; SPGE, screen-printed gold electrode.

b AuNP, gold nanoparticle; NCGF, nanocoral gold foam.

c CV, cyclic voltammetry; DPV, differential pulse voltammetry; EIS, electrochemical impedance spectroscopy.

**Table 3:** A comparison of carbon-based electrochemical biosensors for influenza virus detection

Electrodes^a^	3D surface materials^b^	3D surface materials immobilization method	Capture probes	Capture probe immobilization method	Analytes (influenza subtypes)	Detection methods^c^	Limit of detection	Detection range	Reference
AuSPE	Graphene–Au hybrid nanomaterial	Physical adsorption	Antibody	Electrostatic interaction; chemical conjugation	H3N2	EIS	1.01 μg mL^−1^	1.0–17.5 μg mL^−1^	102
AuSPE	Graphene–Au nanocomposite	Physical adsorption	Glycoprotein	EDC/NHS reaction	H9N2	EIS	10^−8^ U mL^−1^	10^−8^–10^−1^ U mL^−1^	103
GCE	NiO–rGO/MXene nanocomposite	Physical adsorption	Peptide	EDC/NHS reaction	H1N1, H5N1	CV	25–300 nM	2.29–3.63 nM	104
GCE	Graphene–Chi/AuPt NP	Chemical conjugation (glutaraldehyde)	Antibody	Electrostatic interaction; physical adsorption	AIV H9	Amperometry (*I*–*t* curve)	10^0.82^ EID_50_ per mL	10^1.37^–10^6.37^ EID_50_ per mL	105
GCE	CNTs/MoS_*x*_	Physical adsorption	Antibody	EDC/NHS reaction	AIV H7	LSV	1–25 ng mL^−1^	0.43 ng mL^−1^	107

a AuSPE, Au-screen printed electrode; GCE, glassy carbon electrode.

b rGO, reduced graphene oxide; AuPt NP, gold–platinum nanoparticles.

c CV, cyclic voltammetry; EIS, electrochemical impedance spectroscopy; LSV, linear sweep voltammetry.

**Table 4:** A comparison of metal–organic and covalent–organic framework-based electrochemical biosensors for influenza virus detection

Electrodes^a^	3D surface materials^b^	3D surface materials immobilization method	Capture probes	Capture probe immobilization method	Analytes (influenza subtypes)	Detection methods^c^	Limit of detection	Detection range	Reference
Au	polyUiO-66@AgNP	Electrostatic interaction; physical adsorption	Aptamer	Physical adsorption	H1N1	EIS, DPV	54.7 fg mL^−1^	0.1 pg mL^−1^–1 μg mL^−1^	115
GCE	TPB–DVA COFs	Physical adsorption	DNA	Chemical crosslinking (glutaraldehyde)	H1N1	CV, EIS, DPV, CA	5.42 fM	10 fM–1 pM	117
Au	COFs/MWCNT nanocomposites	Physical adsorption	DNA	Chemical crosslinking (glutaraldehyde)	H1N1	DPV	1.01 fM	10 fM–1 nM	118

a GCE, glassy carbon electrode.

b AgNP, silver nanoparticle; TPB–DVA COFs, TPB: 1,3,5-tris (4-aminophenyl) benzene, DVA: 1,4-benzenedicarboxaldehyd, COFs: covalent organic frameworks; MWNTs, multi-walled carbon nanotubes.

c DPV, differential pulse voltammetry; EIS, electrochemical impedance spectroscopy; CV, cyclic voltammetry.

#### 4.2.1 Metal nanoparticles

Metal NPs have gained increasing interest in biosensors due to their excellent electrical, chemical, and physical properties. Table 2 presents representative electrochemical biosensors that have employed metal NPs, listing the types of nanomaterials used and the corresponding detection performance for influenza virus targets. The high surface area, electrical conductivity, and biocompatibility dramatically improve the performance of this class of electrochemical biosensors. These unique properties make metal NPs ideal for immobilizing various biomolecules.

AuNPs are widely used in electrochemical sensors because they can easily be introduced to the electrode surface using various physicochemical methods. The use of AuNPs increases the surface area of the electrode, which improves the immobilization of capture probes such as antibodies and DNA without the loss of bioactivity,^93^ leading to the sensitive detection of influenza viruses in many studies.^94,95^ In a previous study, AuNPs were employed to modify a graphite pencil electrode using simple wet adsorption. A thiol-grouped DNA probe was then covalently immobilized on the AuNPs through the thiol–gold reaction.^95^ EIS and cyclic voltammetry (CV) analysis of the modified recognition surface demonstrated that the incorporation of AuNPs markedly enhanced signal amplification by expanding the effective electrode surface area. The developed sensors successfully detected the influenza B virus, with a detection limit of 54 pM for synthetic target DNA and 3.3 × 10^7^ molecules (in a 30 μL sample) in a real PCR sample.

In addition to AuNPs, zinc oxide (ZnO) nanostructures have also been employed to enhance the performance of electrochemical biosensors.^96^ With a high isoelectric point (∼9.5), ZnO facilitates strong electrostatic interactions with biomolecules that have lower isoelectric points at a physiological pH. This is particularly advantageous for immobilizing antibodies targeting influenza A subtypes such as H1N1, H5N1, and H7N9, which generally have low isoelectric points. To construct 3D nanostructure, ZnO nanorod was grown on the inner surface of PMDS sensor *in situ*. The hydrothermal method facilitates the precise control of the length and diameter of the ZnO, thereby enabling the construction of uniform 3D architectures. As a result, the ZnO surface can produce up to a sixfold increase in antibody loading compared to bare electrodes. Based on this, a ZnO-based multiplexed electrochemical immunosensor has been engineered to simultaneously detect H1N1, H5N1, and H7N9 antigens. The sensor achieved an impressive detection limit of 1 pg mL^−1^, highlighting ZnO's capability to enhance both probe immobilization and sensitivity in influenza virus detection. However, the strong electrostatic properties and high surface area of ZnO may cause non-specific binding of proteins in complex biological samples. In particular, impedimetric sensors, which measure changes at the interface between the electrode surface and the electrolyte solution, can be hindered by such non-specific bindings, resulting in inaccurate measurements.

**Fig. 3:**
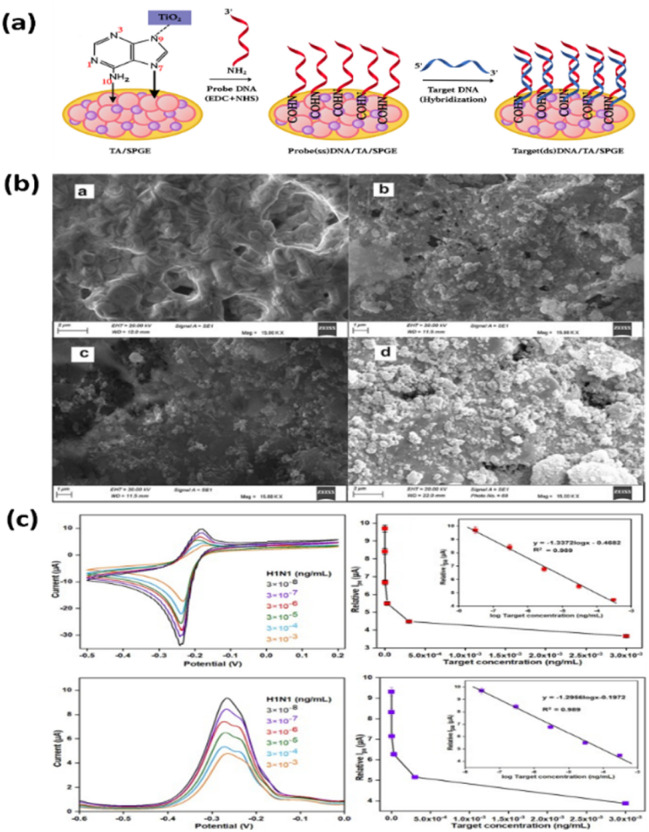
(a) Schematic illustration of the stepwise modification of the SPGE surface, including TiO_2_–adenine nanocomposites, probe (ss)DNA, and target (ss)DNA immobilization. (b) SEM images showing the morphological evolution of the SPGE surface at each modification stage under 150 00× magnification. (c) Electrochemical characterization of the genosensor: CV plots and linear relationship, DPV curves, and peak current response with varying target DNA concentrations. Reproduced from ref. 97 with permission from Elsevier, copyright 2024.

Titanium dioxide (TiO_2_) has also emerged as a valuable component in biosensor platforms due to its large surface area, excellent biocompatibility, and stability. Incorporating TiO_2_ into the electrode surface not only facilitates the effective immobilization of biorecognition elements but also increases the storage capacity of the electrode, leading to improved electrocatalytic performance. Recent studies have employed TiO_2_–DNA nucleotide hybrid nanocomposites to modify the electrode surface, yielding a stronger current response (Fig. 3).^97,98^ This improvement has been attributed to more efficient electron transport and a 3D network structure that increased the effective surface area. Because of this nanostructured interface, a fabricated biosensor has achieved sensitive and reliable detection of the H1N1 influenza virus with high sensitivity (10.6 μA ng^−1^ cm^−2^), a low detection limit of 6.7 × 10^−8^ ng mL^−1^, and a wide linear range of 3 × 10^−8^–3 × 10^−3^ ng mL^−1^, highlighting the potential of TiO_2_-based hybrid materials for advancing viral diagnostics.

**Fig. 4:**
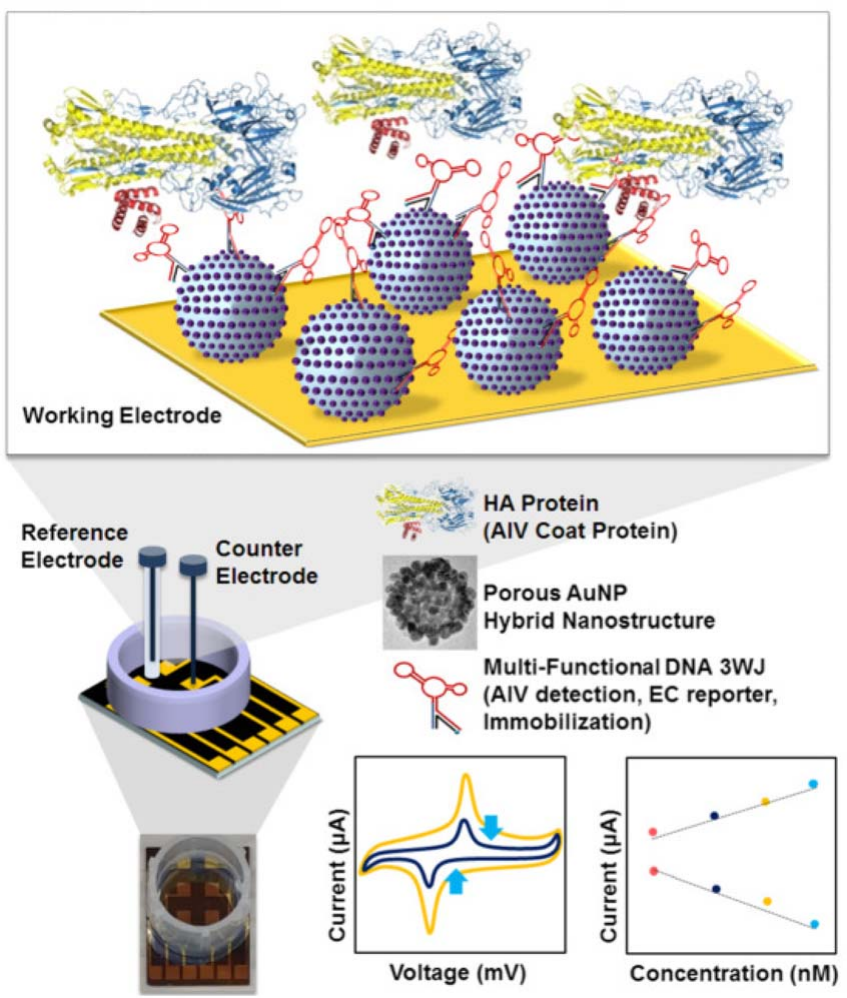
Schematic image of the fabricated AIV detection biosensor based on porous AuNP-modified electrodes. Reproduced from ref. 100 with permission from Elsevier, copyright 2019.

In addition to the use of innovative metal NPs, structural engineering of the electrode surface, especially by introducing porosity, has become a key approach to enhancing biosensor performance. Porous electrodes offer a higher specific surface area and direct channels that facilitate improved diffusion and more efficient electrolyte transport within the electrode.^99^ For example, numerous studies have explored strategies to impart porosity on AuNPs, aiming to increase their surface area and boost the sensitivity and overall effectiveness of biosensors. Because porous AuNPs promote electron transfer and immobilize more recognition elements than bare AuNPs due to improved surface roughness and high adsorption, porous AuNP-treated biosensors have demonstrated greater sensitivity and stronger electrochemical signals (Fig. 4).^100^ In addition, nano-coral Au foam (NCGF) has been utilized on an electrochemical biosensor to detect influenza virus A.^101^ The nano-coral structure with interconnected micro- and nano-pores significantly increased the surface area, thereby improving the performance of electrochemical immunosensors. Compared to the bare AuNPs and gold nanoflower-treated biosensors, the developed sensor exhibited a higher peak current and peak area. These results illustrate that electrodes with a hierarchical structure and tailored surface porosity facilitate molecular diffusion and optimize reaction kinetics. Due to the large surface area of NCGF, NCGF-based biosensors are able to detect influenza A virus sensitively, with a LOD of 13.14 fg mL^−1^, which was 10 times higher than conventional electrochemical immunosensors.

#### 4.2.2 Carbon-based materials

Carbon-based materials such as activated carbon, graphene, and carbon nanotubes (CNTs) have received significant interest for the development of electrochemical biosensors due to their superior electrical conductivity, large surface area, and outstanding stability. Notably, composites incorporating these carbon materials have demonstrated strong potential in high-precision analytical applications, including virus detection.^102,103^ A summary of key studies is presented in Table 3.

Graphene and chemically modified graphene sheets (*e.g.*, rGO) are prominent examples of 2D carbon-based materials known for their high electrical conductivity, surface area, and mechanical properties. As discussed in Section 4.1, graphene oxide-modified electrodes exhibited improved sensitivity due to their higher surface roughness and effective surface area. Several studies have explored hybrid composites that combine graphene with NPs to improve biosensor performance, harnessing their synergistic effects to construct a 3D electrode architecture that promotes greater biomolecule immobilization and more efficient signal transduction. In particular, graphene modified with AuNPs represents a fundamental design for electrochemical biosensors that target the rapid and sensitive detection of influenza viruses.^102,103^ The excellent electrical conductivity and biocompatibility of AuNPs, combined with the wrinkled 3D structure of the electrode surface, significantly enhance the sensor's sensitivity.

**Fig. 5:**
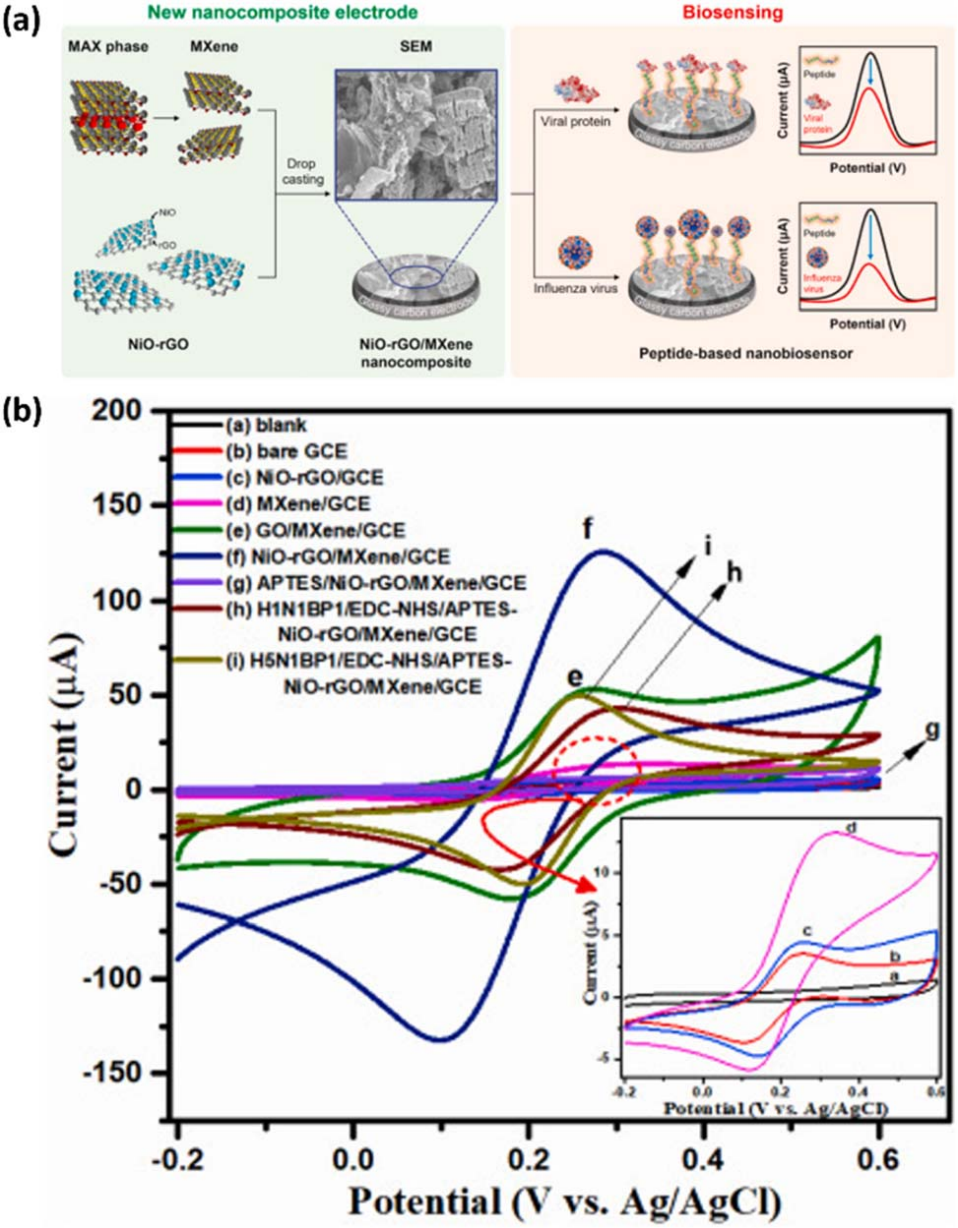
(a) Schematic overview of the peptide-based nanobiosensor fabrication and detection strategy for influenza virus and viral proteins, based on a 3D hybrid NiO–rGO/MXene nanostructure (b) CV analysis of various electrode modifications, highlighting the impact of nanocomposite and biomolecule assembly on electrochemical behavior. Reproduced from ref. 104 with permission from Elsevier, copyright 2022.

In another study, nanocomposite bioelectrodes composed of nickel oxide (NiO) and rGO were fabricated to develop highly efficient peptide-based electrochemical biosensors (Fig. 5).^104^ Combining NiO nanostructures with rGO resulted in a mesoporous composite with a high pore volume, accommodating more bioreceptors. This increased bioreceptor density facilitated the capture of more target antigens, significantly improving the biosensor's sensitivity. In addition, MXenes have emerged as a promising nanomaterial for electrochemical sensors because of their hydrophilic nature and excellent electrical conductivity. However, the use of small-sized MXene flakes can introduce resistance. To address this, a hybrid structure combining a MXene with graphene was employed. This approach resulted in a NiO–rGO/MXene composite that significantly enhanced electron transport and provided a large active surface for effective peptide attachment and virus detection. As a result, the NiO–rGO/MXene composite could significantly improve electron transfer and provide a large surface area for peptide immobilization and virus capture. This biosensor demonstrated outstanding sensitivity in detecting H5N1 and H1N1 HA antigens in a buffer solution, achieving detection limits of 2.29 and 3.09 nM, respectively. These results indicate that integrating graphene with NPs in a hybrid nanostructure allows for highly sensitive influenza virus detection due to the large surface area of graphene and the unique functional properties of the NPs.

The high surface area of graphene not only supports the effective immobilization of capture probes but also allows for the attachment of detection probes, leading to improved sensitivity and stronger signal outputs. Accordingly, research has progressively shifted from concentrating solely on capture probe immobilization and signal transduction to emphasizing signal amplification techniques. For example, a graphene–Au/Pt complex has been used in an enzyme-free sandwich-type electrochemical immunosensor for the effective detection of avian influenza virus H9, which is responsible for significant economic losses.^105^ Because Au and Pt NPs have excellent electrochemical catalytic properties, they were used to enhance the sensitivity of the electrochemical immunosensor. The graphene matrix platform had a large surface area and offered numerous active sites for the attachment of multiple Au/Pt NPs, significantly enhancing the conductivity and catalytic performance. These Au/Pt NPs served as modified active sites for labeling and immobilizing avian influenza virus H9 polyclonal antibodies, amplifying the detection signal. Due to the excellent catalytic properties, superior conductivity of graphene and NPs, and effective antibody immobilization, the sensor achieved a high sensitivity of 10^0.82^ EID_50_ per mL and strong reproducibility.

CNTs are cylindrical structures formed by rolling graphene sheets, and they can be classified as single- or multi-walled depending on the number of graphene layers. As with carbon-based materials, CNTs have a large surface area and excellent electrical conductivity. For this reason, multi-walled CNTs (MWCNTs) are commonly utilized to modify the electrode surface.^106,107^ MWCNT-based electrodes have a porous structure with a large effective surface area, high electrocatalytic activity, and electronic conductivity with enhanced charge transfer channels. Building on these advantages, a novel hybrid material has been developed by integrating MWCNTs with molybdenum sulfide (MoS_*x*_) *via* a solvothermal method, resulting in a 3D CNT/MoS_*x*_ aerogel.^107^ CNT/MoS_*x*_ formed an interconnected porous network that increased the electroactive surface area and electron transfer efficiency, offering ideal conditions for biosensing applications. The CNT/MoS_*x*_ aerogel was utilized to construct an electrochemical immunosensor to detect avian influenza H7 virus. The 3D porous structure of CNT/MoS_*x*_ provided optimal conditions for the immobilization of capture and detection antibodies, increasing the interaction with the target virus. The developed biosensor exhibited a linear detection range of 1–25 ng mL^−1^ and a detection limit of 0.43 ng mL^−1^, demonstrating high sensitivity and reliability in detecting and quantifying avian influenza H7.

However, despite the advantages, certain factors must be considered when using carbon-based materials in the fabrication of 3D architecture. Carbon-based material easily forms aggregates in aqueous solution due to the hydrophobic π–π interaction between individual materials.^108^ These aggregations can reduce the specific surface area and lower the conductivity.^109^ In addressing this issue, studies have been reported that either increase the interplanar space by using spacer materials or reduce aggregation by developing hybrid materials that combine carbon nanotubes and graphene nanosheets.^110–112^ Further research is still required to resolve this issue.

#### 4.2.3 Metal–organic and covalent–organic framework-based materials

**Fig. 6:**
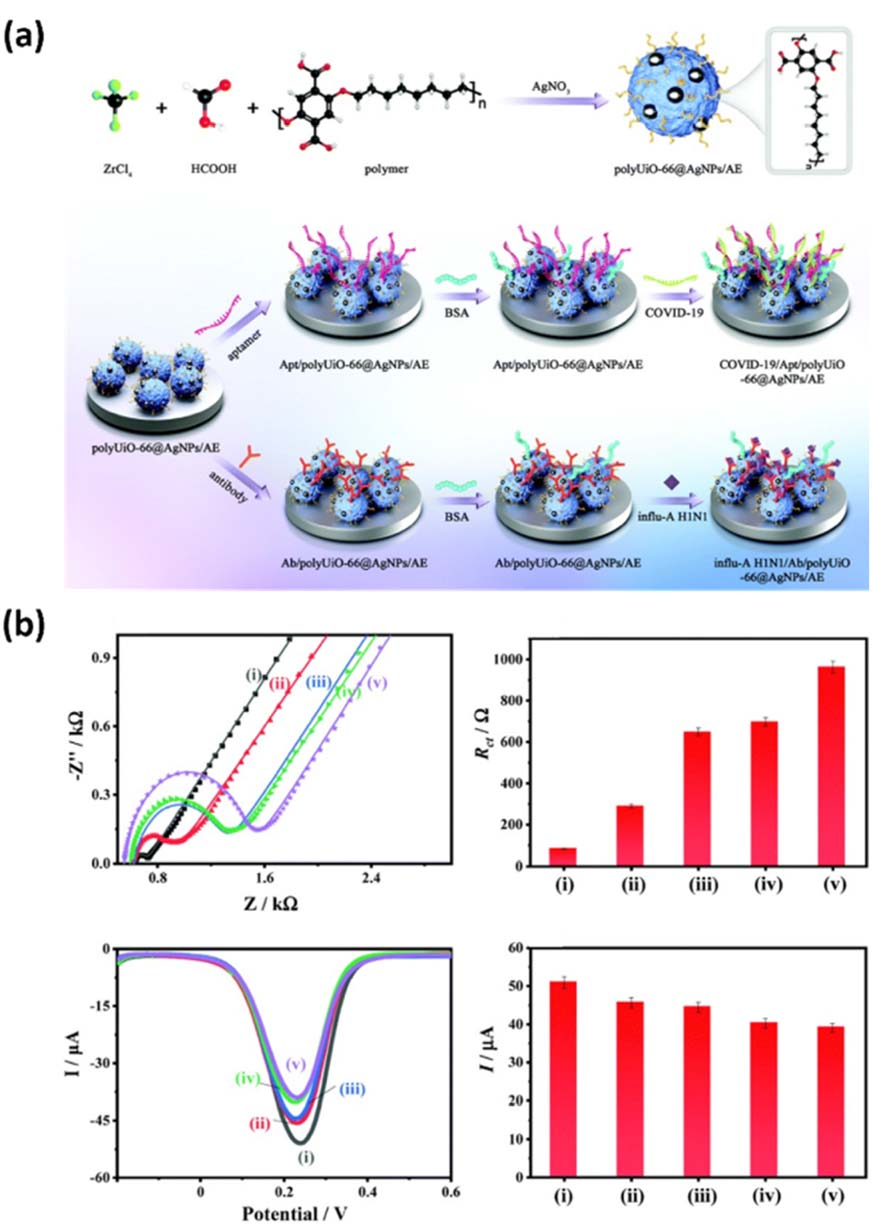
(a) Preparation of polyUiO-66@AgNPs and construction of polyUiO-66@AgNP-based biosensors for the detection of H1N1 and N-gene of SARS-CoV2. (b) Electrochemical performance of polyUiO-66@AgNPs-based biosensors for H1N1 detection, showing EIS Nyquist plots, charge transfer resistance, DPV responses, and peak currents. Reproduced from ref. 115 with permission from the Royal Society of Chemistry, copyright 2021.

Organic frameworks are crystalline porous materials with a high surface area, facilitating efficient transportation and storage.^113^ An overview of recent electrochemical biosensors employing metal–organic framework (MOF)- and covalent–organic framework (COF)-based materials is provided in Table 4. An MOF is composed of metal ions and organic ligands that act as nodes and linkers, respectively. MOFs can be engineered to include pores on a micro- to meso-scale, offering numerous active sites for the attachment of various probes.^114^ Their strong affinity for biomolecules also enhances probe adsorption, making them well-suited for biosensing applications. The zirconium-based MOF hybrid material polyUiO-66, has been designed by introducing a polymer as a spacer within a MOF network (Fig. 6).^115^ Adjusting the polymer length allowed for precise control over the pore size, enabling the incorporation of NPs into the pores. These NPs enhanced the conductivity and sensitivity of the resulting electrochemical biosensor. With its high porosity, diverse functional groups, and interconnected network structure, polyUiO-66 was well-suited for encapsulating silver NPs (AgNPs), forming the polyUiO-66@AgNP composite. This material exhibited excellent electrochemical properties and a strong affinity for antibodies and aptamers. The high surface area, large pore size, and abundant surface functional groups of polyUiO-66@AgNP immobilized many H1N1 antibodies. In particular, combining the outstanding electrochemical activity and biocompatibility of AgNPs with the high porosity and rich functionality of polyUiO-66 led to a substantial enhancement in signal amplification and selective target recognition capabilities. The developed biosensor exhibited an extremely low LOD of 54.7 fg mL^−1^ for the H1N1 virus, along with high selectivity, reproducibility, stability, and reusability. This study thus represents a new direction for the development of electrochemical biosensors using hybrid polymer–MOF materials, demonstrating their potential as a platform for the sensitive and selective diagnosis of respiratory viruses.

Recently, COFs have gained attention for use in electrode fabrication. Compared to MOFs, COFs have greater potential as electrode materials due to their adjustable π-conjugated structure, which improves conductivity, exceptional thermal and chemical stability, which maintains structural integrity in demanding electrochemical environments, and a high degree of functional versatility.^116^ A COF based on 1,3,5-tris(4-aminophenyl)benzene (TPB) and 1,4-benzenedicarboxaldehyde (DVA) has been synthesized using a Schiff base reaction.^117^ The TPB-DVA-based COF had a unique 3D pom-pom-like structure with a rough surface texture, offering a high specific surface area of 554.7 m^2^ g^−1^ and an average pore diameter of 2.19 nm. The high surface area, attributed to the natural porosity of the COF structure, facilitated the effective immobilization of capture probes and boosted the efficiency of redox reactions and electron transfer at the electrode surface. The resulting TPB-DVA COF-based biosensor enabled the sensitive detection of multiple influenza A virus subtypes, including H1N1, H5N1, H5N6, and H7N2, with a detection range of 0.1 pM to 20 pM and an LOD of 0.026 pM, demonstrating its potential for broad-spectrum virus biosensing.

**Fig. 7:**
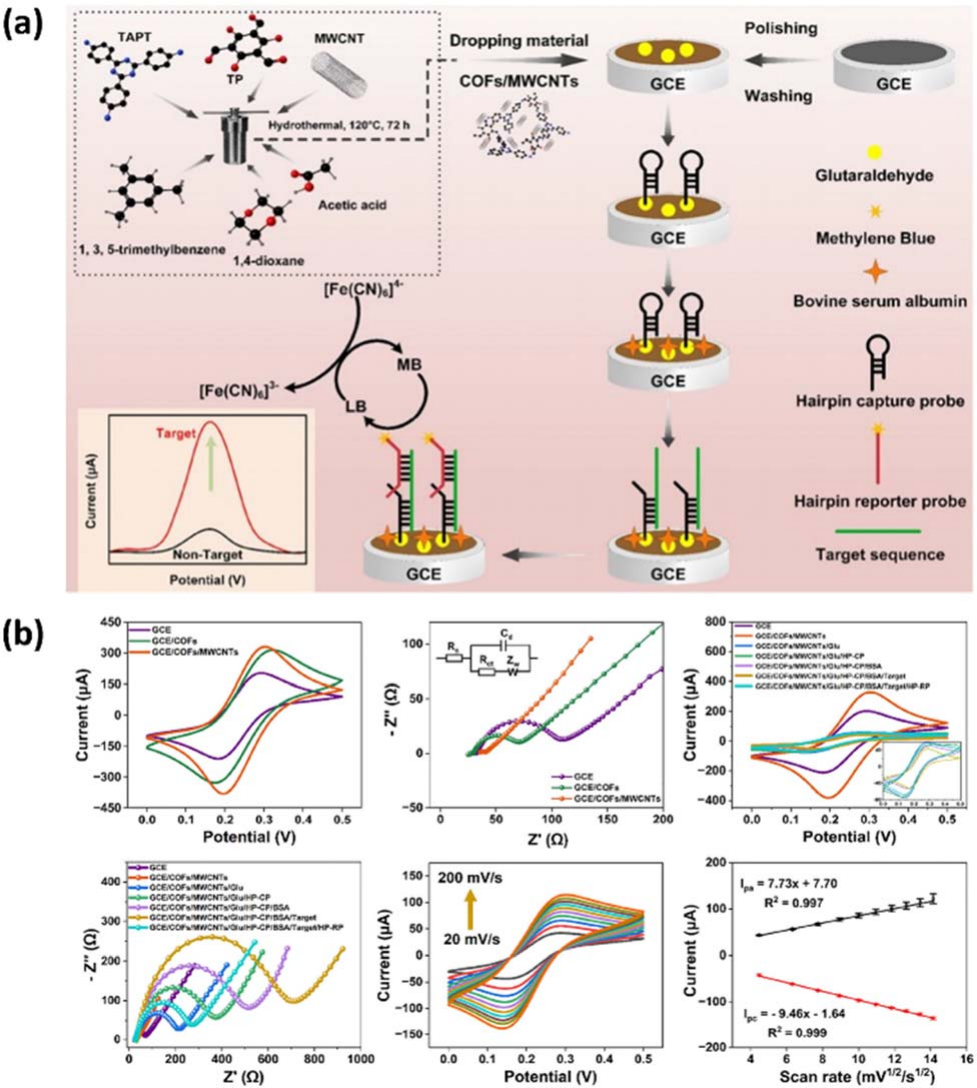
(a) Schematic diagram of the construction process of the electrochemical biosensor based on a COF/MWCNT porous nanocomposite. (b) Electrochemical characterization of COFs and COFs/MWCNTs-modified electrodes through cyclic voltammetry and impedance spectroscopy, including scan rate-dependent analysis. Reproduced from ref. 118 with permission from American Chemical Society, copyright 2024.

To further enhance biosensor sensitivity, an electrochemical biosensor was developed by combining COFs and MWCNTs for the rapid and sensitive detection of complementary DNA (cDNA) related to the H1N1 virus (Fig. 7).^118^ The COF/MWCNT composite enhanced the performance of the electrochemical biosensor by combining the porous structure and chemical tunability of the COF with the excellent conductivity and mechanical stability of the MWCNTs.^119^

A dual-probe system was designed using the COFs/MWCNT composite, incorporating 4,4′,4′′-(1,3,5-triazine-2,4,6-triyl)trianiline and 1,3,5-triformylphloroglucinol to enable the precise detection of H1N1 virus cDNA.^118^ N_2_ adsorption–desorption isotherm and BET analysis revealed that it has an increased surface area of 783.3 m^2^ g^−1^ compared to the aforementioned TPB-DVA-based COF, and features pores with a micropore size of 0.53 nm. Based on the electrochemical signals produced through the interaction between the COF and MWCNT complex, the biosensor achieved a broad detection range from 10 fM to 1 nM and an impressively low LOD of 1.01 fM. Moreover, the sensor was also used in a portable device, achieving a detection limit as low as 0.17 fM, with an accuracy of over 98% in spiked recovery experiments, thus producing results that closely matched those obtained through droplet digital PCR in mouse tissue samples. The microporous structure of COF/MWCNTs has been proven to play a crucial role in enhancing the sensitivity of biosensors by increasing both the surface area and ion storage capacity. However, if the pore size is insufficient, the access of certain large ions may be restricted, thereby increasing the dead volume of the electrode.^120^ Consequently, the efficacy of the biosensor can be hindered by the size of the ions and the type of electrolytes used. In particular, potentiometric biosensors, which measure the potential dependent on ion concentration, have limitations in their application.

## 5 Conclusion

This review summarized the development of recent electrochemical biosensors that use 3D surface-coating materials for the sensitive and selective detection of influenza viruses. Incorporating 3D structures into these biosensors enhances their analytical performance by increasing the available surface area for probe attachment, thereby improving sensitivity and lowering the LOD. A variety of functional materials, including metal nanoparticles, carbon-based materials, MOFs, and COFs, have been utilized to modify these biosensors for the detection of specific influenza virus strains. Numerous studies have evaluated their performance using clinical samples, and successful detection of influenza viruses has been reported.^94,98,101,105^ Furthermore, recent trends, such as portable and smartphone-integrated devices^101^ and wearable biosensors,^121^ are broadening the scope of point-of-care testing (POCT) and real-time monitoring.

Despite these advancements, several challenges remain for their clinical application. Depending on the type of sensor and the characteristics of the capture probe, complex sample matrices may require additional pretreatment steps, which can limit their immediate applicability in POCT environments. In the case of wearable biosensors, the biocompatibility of 3D materials becomes a critical consideration, which was relatively less important in conventional *in vitro* diagnostics. Therefore, further optimization of sensor components, surface functionalization strategies, and probe design is required to enhance detection efficiency, reduce production costs, ensure biocompatibility, and enable practical implementation. Continued research and development in this field is critical to improving the clinical applicability of 3D-structured electrochemical biosensors, which could strengthen influenza virus monitoring and enable rapid diagnosis, ultimately contributing to improved public health responses.

## Author contributions

HWK: writing – original draft, resources, methodology, data curation. ASL: writing – original draft, resources, methodology, data curation. CSK: writing – review & editing, writing – original draft, supervision, conceptualization.

## Conflicts of interest

There are no conflicts to declare.

## Data Availability

No primary research results, software, or code have been included and no new data were generated or analyzed as part of this review.
